# Development and validation of a prognostic model based on endoplasmic reticulum stress-related lncRNAs in breast cancer

**DOI:** 10.3389/fonc.2025.1613938

**Published:** 2026-01-12

**Authors:** Mingfei Chu, Xialing Shi, Baojun Huang, Mengfei Chen

**Affiliations:** 1 Department of Surgical Oncology and General Surgery, The First Hospital of China Medical University, Shenyang, China; 2 Department of Sterile Supply, The First Affiliated Hospital of Shandong Second Medical University, Weifang People’s Hospital, Weifang, China

**Keywords:** breast cancer, endoplasmic reticulum stress-related long non-coding RNAs, LMNTD2-AS1, oncology, prognostic model

## Abstract

**Background:**

Breast cancer (BC), recognized as the most prevalent malignant tumor among women, has emerged as a critical global public health concern. Increasing evidence indicates that long non-coding RNAs (lncRNAs) are essential regulators in the initiation, development, and progression of BC, influencing tumor biology through diverse molecular mechanisms. Despite these advances, the specific prognostic significance of endoplasmic reticulum (ER) stress (ERS)-related lncRNAs in BC remains largely unexplored, and no comprehensive study has yet been reported in this context.

**Methods:**

In this study, we utilized high-throughput BC data from the TCGA database to identify ERS-related lncRNAs that are strongly associated with patient survival and prognosis. Based on these findings, we developed a risk model consisting of six ERS-related lncRNAs within the TCGA cohort, which demonstrated independent prognostic value for BC patients. We further examined the association of this model with clinical outcomes, the tumor microenvironment (TME), immune cell infiltration, and the predictive potential of immune checkpoint expression. In addition, we conducted functional analyses to explore the signaling pathways and biological processes regulated by ERS-related lncRNAs, providing new insights into their role in BC progression and prognosis.

**Results:**

The prognostic model constructed from six ERS-related lncRNAs demonstrated strong predictive performance, with AUC values of 0.702, 0.707, and 0.676 for estimating survival at 1, 3, and 5 years, respectively. Kaplan-Meier survival analyses further revealed that patients in the high-risk group exhibited significantly poorer overall survival (OS) compared to those in the low-risk group. Gene set enrichment analysis (GSEA) indicated that the prognostic model may be functionally linked to pathways involving the extracellular matrix (ECM) and focal adhesions. Among the six ERS-related lncRNAs, LMNTD2-AS1 was selected for functional validation, and experimental results demonstrated that its knockdown suppressed the proliferation, migration, and invasion abilities of breast cancer cells.

**Conclusions:**

Overall, our ERS-related lncRNA risk signature not only provides valuable prognostic insights but may also serve as a potential therapeutic target and biomarker to improve the management and treatment strategies for BC patients.

## Introduction

Breast cancer (BC) has emerged as the most prevalent malignancy among women, representing a major public health concern due to its steadily rising incidence. According to the latest statistics from the international agency for research on cancer (IARC), more than 2.3 million new BC cases were reported worldwide in 2023, accounting for 11.7% of all malignant tumors (1–3). Based on the expression of receptors, estrogen receptor (ER), progesterone receptor (PR), and human epidermal growth factor receptor 2 (HER2), BC is classified into three major subtypes: hormone receptor-positive, HER2-positive, and triple-negative BC (TNBC) (4). HER2-positive BC is a subtype defined by overexpression or amplification of the human HER2 gene, accounting for approximately 20% to 30% of all BC cases (5). Hormone receptor-positive BC refers to cases in which the cancer cells test positive for the expression of ER or PR on their surface (6). TNBC is a highly aggressive subtype of BC characterized by the lack of expression of ER and PR, along with low or absent expression of HER2 (7). Standard therapeutic strategies include mastectomy, chemotherapy, endocrine therapy, radiotherapy, and targeted therapy. Immunotherapy is increasingly recognized as an important therapeutic strategy for TNBC, attracting growing attention. Neoantigens (neoAgs) and cancer-testis antigens (CTAs) serve as tumor-specific targets, originating from somatic mutations and epigenetic alterations in cancer cells. These antigens offer promising prospects for the development of personalized cancer vaccines (8). Despite remarkable advances in early screening and comprehensive treatment approaches, the multifactorial pathogenesis of BC and the emergence of treatment resistance continue to present significant challenges in clinical management (9). With the rapid development of high-throughput sequencing, multi-omics technologies, and advanced big data analytical methods, the paradigm of BC research has shifted from conventional histopathological subtyping toward multidimensional molecular profiling. In this context, the identification of novel biomarkers is essential for unraveling the molecular mechanisms driving BC progression and for improving prognosis and therapeutic outcomes.

The endoplasmic reticulum (ER), the largest subcellular organelle, performs several essential biological functions, including co-translational protein folding, regulation of calcium ion (Ca^2+^) homeostasis, and steroid biosynthesis (10). These processes can be disrupted by various external stressors or intrinsic cellular events, impairing the ER’s protein-folding capacity and leading to the accumulation of misfolded or unprocessed proteins (11). When the ER’s ability to correctly fold proteins or degrade defective ones is overwhelmed, misfolded proteins accumulate and trigger a harmful condition known as ER stress (ERS), which compromises cellular function and survival (12). ERS has been closely linked to tumorigenesis and chemotherapy resistance, as the ability of cancer cells to adapt to persistent ERS promotes survival, angiogenesis, metastatic progression, drug resistance, and immune evasion. In BC, ERS plays a pivotal role in sustaining proteostasis and supporting tumor evolution, making it a potential therapeutic target (13). Evidence also suggests that ERS is involved in prostate cancer pathogenesis, where the unfolded protein response (UPR) interacts with androgen receptor signaling, further highlighting its role in cancer biology (14). A deeper understanding of how tumor cells exploit ERS mechanisms to their advantage could uncover critical vulnerabilities, and exploring the association between ERS and carcinogenesis carries significant implications for developing novel therapeutic strategies.

Long non-coding RNAs (lncRNAs), a class of transcripts longer than 200 nucleotides that do not encode proteins, play crucial roles in diverse cellular processes including transcription, translation, stem cell differentiation, autophagy, apoptosis, and epigenetic regulation (15–17). Alterations in lncRNA expression have been strongly associated with malignant tumors, underscoring their dual roles as potential oncogenes or tumor suppressors in cancer biology (18, 19). Through interactions with DNA, lncRNAs regulate transcription, epigenetic modifications, RNA and protein stability, translation, and post-translational modifications, while some studies also suggest that lncRNAs can directly interact with specific signaling receptors (20, 21). Among the well-characterized lncRNAs, PVT1 has been shown to significantly influence the initiation and progression of BC, highlighting its potential as both a diagnostic biomarker and a therapeutic target. Acting as a driver of cancer cell proliferation, invasion, and metastasis, PVT1 has emerged as an attractive candidate for therapeutic intervention, while also providing valuable insights for diagnostic research in BC (22). HOTAIR is a carcinogenic non-coding RNA, whose expression level is significantly correlated with the grading and prognosis of various malignant tumors including BC. It regulates multiple target genes through sponge adsorption mechanisms and epigenetic mechanisms, thereby influencing key carcinogenic cellular processes and signaling pathways such as tumor metastasis and drug resistance (23). Upregulation of HOTAIR has been linked to BC progression, suggesting it as another promising therapeutic target (24). Collectively, identifying and characterizing lncRNAs associated with BC may greatly enhance our understanding of the molecular mechanisms underlying tumor progression, while also advancing biomarker discovery, therapeutic development, and strategies to overcome chemotherapy resistance (25).

In this study, we analyzed BC RNA sequencing data from the TCGA database to identify ERS-related lncRNAs significantly associated with patient survival and prognosis. Based on these findings, we developed a prognostic risk model consisting of six ERS-related lncRNAs within the TCGA cohort, which served as an independent prognostic factor for BC patients. Subsequent analyses examined the correlation of this model with clinical outcomes, tumor microenvironment characteristics, immune cell infiltration, and the predictive potential of immune checkpoint expression. Furthermore, we explored the signaling pathways and biological processes regulated by ERS-related lncRNAs, providing insights into their functional relevance in BC progression. Among the six ERS-related lncRNAs identified, the role of LMNTD2-AS1 in BC has not been previously reported. To address this gap, we sought to investigate its influence on malignant biological behaviors, including cell proliferation, migration, and invasion. By silencing LMNTD2-AS1 in BC cells, we demonstrated its significant impact on these processes, thereby providing new evidence of its functional role in tumor biology. These findings offer valuable insights into the molecular mechanisms driving BC development and progression, highlighting LMNTD2-AS1 as a potential target for future therapeutic research.

## Materials and methods

### Data acquisition and ERS-related lncRNAs identification

We downloaded transcriptome profiles (RNA-Seq) of 1,035 human breast tissue samples from the TCGA database (https://portal.gdc.cancer.gov), including 941 BC samples and 94 normal samples. ERS-related genes were obtained from GeneCards databases (https://www.genecards.org/), and corresponding clinical data for BC samples were also collected from TCGA. To identify candidate lncRNAs, ERS-related lncRNAs were first screened using the limma package in R software based on a Pearson correlation coefficient threshold of |cor|>0.6, *P* < 0.001. Subsequently, ERS-related differentially expressed lncRNAs were identified for further analysis using the criteria of |log2 fold change (FC)| > 1 and false discovery rate (FDR) < 0.05.

### Construction and verification of ERS-related risk model

For this study, the entire dataset was randomly divided into training and testing sets in a 1:1 ratio, with 50% of the samples allocated to each group. Model construction was performed using the training cohort, while the testing cohort was employed to validate the model’s performance. Within the training set, an ERS-related lncRNA prognostic signature was established using multivariate Cox proportional hazards regression analysis. Cox regression analysis, as a multivariate analysis method, is particularly suitable for analyzing survival data. It can adjust for confounding factors, thereby enabling comparisons between groups, and allows for multivariate survival prediction. Its core lies in incorporating the time factor to perform regression analysis. Based on the median risk score as the cutoff value, patients were stratified into high-risk and low-risk groups, and differences in prognostic outcomes between the two groups were evaluated using Kaplan-Meier survival curves. The predictive accuracy of the model was further assessed in the testing set by calculating the area under the ROC curve (AUC) with R software. The risk score for each BC patient was calculated according to the following formula.


RiskScore =∑k=1n(Coefk×Expk)

Exp(k) represents the relative expression level of each ERS-related lncRNA, while Coef(k) denotes the corresponding regression coefficient derived from the multivariate Cox regression analysis.

### Construction of the nomogram for BC patients

We collected and analyzed the clinical data of BC patients, including age, clinical stage, TNM stage, and risk scores, to construct a prognostic nomogram using the regplot, survival, and rms packages in R for predicting 1-, 3-, and 5-year overall survival (OS). Calibration curves were then generated to evaluate the consistency between the actual survival outcomes and the model’s predicted prognosis.

### Differentially expressed gene and functional analysis

DEGs between the high-risk and low-risk groups were identified using the criteria of |log2 FC| > 1 and a FDR < 0.05. The ERS-associated genes obtained from expression analysis were further subjected to gene ontology (GO) and KEGG enrichment analyses using the ClusterProfiler R package to identify significantly enriched biological processes and pathways. In addition, gene set enrichment analysis (GSEA) and related approaches were employed to explore signaling pathways and biological processes associated with ERS-related genes across the high- and low-risk subgroups.

### Relevance and survival analysis of tumor mutation load

To determine whether the risk score was associated with TMB, we conducted a correlation analysis using the ggpubr and reshape2 R packages. The resulting scatter plot indicated that a correlation coefficient (R) < 0 reflected a negative association, while the opposite held true when R > 0, with statistical significance set at *P* < 0.05. Based on TMB levels, patients were stratified into high- and low-mutation load groups, and survival analyses were performed accordingly. Furthermore, an integrated Kaplan-Meier survival analysis was conducted across four subgroups, high TMB + high risk, high TMB + low risk, low TMB + high risk, and low TMB + low risk, to evaluate the combined impact of TMB and risk score on patient prognosis.

### Tumor microenvironment and immune cell infiltration analysis

The analysis of the tumor microenvironment (TME) was used to assess tumor purity in BC patients. The ESTIMATE (Estimation of Stromal and Immune cells in Malignant Tumors using Expression data) algorithm is a quantitative method that utilizes gene expression data from cancer tissues to estimate the stromal and immune cell infiltration within the tumor microenvironment. By analyzing specific signals in gene expression patterns, the ESTIMATE algorithm generates three scores: the Stromal Score (representing the abundance of stromal cells), the Immune Score (reflecting the abundance of immune cells), and the ESTIMATE Score (indicating the overall concentration of non-tumor cellular components in the tumor microenvironment). These scores help researchers assess the composition of the tumor microenvironment, thereby enabling a better understanding of tumor behavior and the design of targeted therapeutic strategies. Immune score, stromal score, and estimate score were calculated using the estimate R package, followed by comparative analysis to evaluate differences in TME features between high- and low-risk groups. To explore the association between immune cells and risk scores, immune cell correlation analysis was performed using ggplot2, ggtext, tidyverse, and ggpubr R packages. Survival analysis of immune cell infiltration was then carried out with the limma, survival, and survminer R packages, and results were filtered at *P* < 0.05 before plotting Kaplan-Meier curves. In addition, single-sample GSEA (ssGSEA) was conducted using the GSVA and GSEABase R packages to examine immune cell scores and related functional pathways.

### Cell lines and transfection

In this study, we utilized the human normal mammary epithelial cell line MCF10A along with five BC cell lines: BT-474, MCF-7, MDA-MB-231, T-47D, and SKBR-3. All cells were cultured in RPMI-1640 medium (Pricella, China) supplemented with 10% fetal bovine serum (FBS, Gibco, New Zealand) and 1% penicillin-streptomycin (Biosharp, China) at 37 °C in a humidified atmosphere containing 5% CO_2_. For transfection, shRNA plasmids (JTS Scientific, China) were employed, and the procedure was carried out according to the manufacturer’s recommended plasmid dosage once cell confluence reached 40-50%. The shRNA sequences used were as follows:sh-NC:5’-UUCUCCGAACGUGUCACGUTT-3’, 5’-ACGUGACACGUUCGGAGAATT-3’, sh-LMNTD2-AS1#1: 5’-GUGAUGACAGCGCACGUUATT-3’, 5’-UAACGUGCGCUGUCAUCACTT-3’; sh-LMNTD2-AS1#2:5’-CCAUUACCAUGACAAGUAUTT-3’, 5’-AUACUUGUCAUGGUAAUGGTT-3’.

### RNA detection and quantitative real-time PCR

Total RNA was extracted using TRIzol reagent (Invitrogen, USA) according to standard protocols, and RNA purity and concentration were determined with a NanoDrop spectrophotometer. Reverse transcription was carried out using the HiScript II 1st Strand cDNA Synthesis Kit (Vazyme, Jiangsu, China) following the manufacturer’s instructions, with reactions prepared in RNase-free EP tubes. The resulting cDNA was immediately subjected to qRT-PCR analysis, and gene expression levels were quantified using the 2^−^ΔΔCt method. The primers used in this study were as follows: LMNTD2-AS1: forward, AGTGACAGGCACTCACCTACCT; reverse, CTGGAGCAGAGGGAATACTTGT GAPDH: forward, CAAGGCTGTGGGCAAGGTCATC; reverse, GTGTCGCTGTTGAAGTCAGAGGAG.

### Wound healing and colony formation assay

After completing cell transfection, the cells were seeded into 6-well plates, and once the cell density reached approximately 90%, scratch wounds were created using a 200 μl pipette tip guided by a ruler to ensure consistency. Images of the wound areas were captured at 48 hours post-scratching using an inverted microscope (Nikon, Japan).

Log-phase transfected cells were digested, centrifuged, and seeded into six-well plates at a density of 2,000 cells per well. After 2–3 weeks, colonies were fixed with 4% paraformaldehyde (Biosharp, China), stained with crystal violet (Solarbio, China), washed, and air-dried. Images of the colonies were captured using a stereomicroscope, and colony formation efficiency was calculated using the formula: (number of colonies/number of seeded cells) × 100%.

### Migration and invasion assay

Corning Matrigel was thawed overnight at 4 °C, diluted, and plated onto the upper surface of Transwell inserts (Corning, USA), followed by incubation for 1 hour to allow solidification into a thin layer. After removing the excess medium, transfected cells were suspended in serum-free medium, and 100 μL of this suspension containing 10,000 cells was added to the upper chambers. The lower chambers were filled with 500 μL of medium supplemented with 10% FBS (FBS, Gibco, New Zealand) as a chemoattractant. Cells were incubated in the upper chambers for 24 hours, after which non-invasive cells were carefully removed, while the remaining cells were fixed with 4% paraformaldehyde (Solarbio, China), washed with PBS, and stained with crystal violet (Solarbio, China). Five randomly selected fields were observed under an inverted microscope, and representative images were captured. Migration assays were performed using non-Matrigel-coated inserts, whereas invasion assays were conducted with Matrigel-coated wells.

### Statistical analysis

Statistical analyses were performed using SPSS 19.0 and GraphPad Prism 9.0, while clinical data and RNA sequencing data were processed with R (version 4.0.2) and Perl (version 5.30.0.1). For all evaluations, a P-value < 0.05 was considered statistically significant.

## Results

### Identification and classification of ERS-related lncRNAs

**Figure 1:**
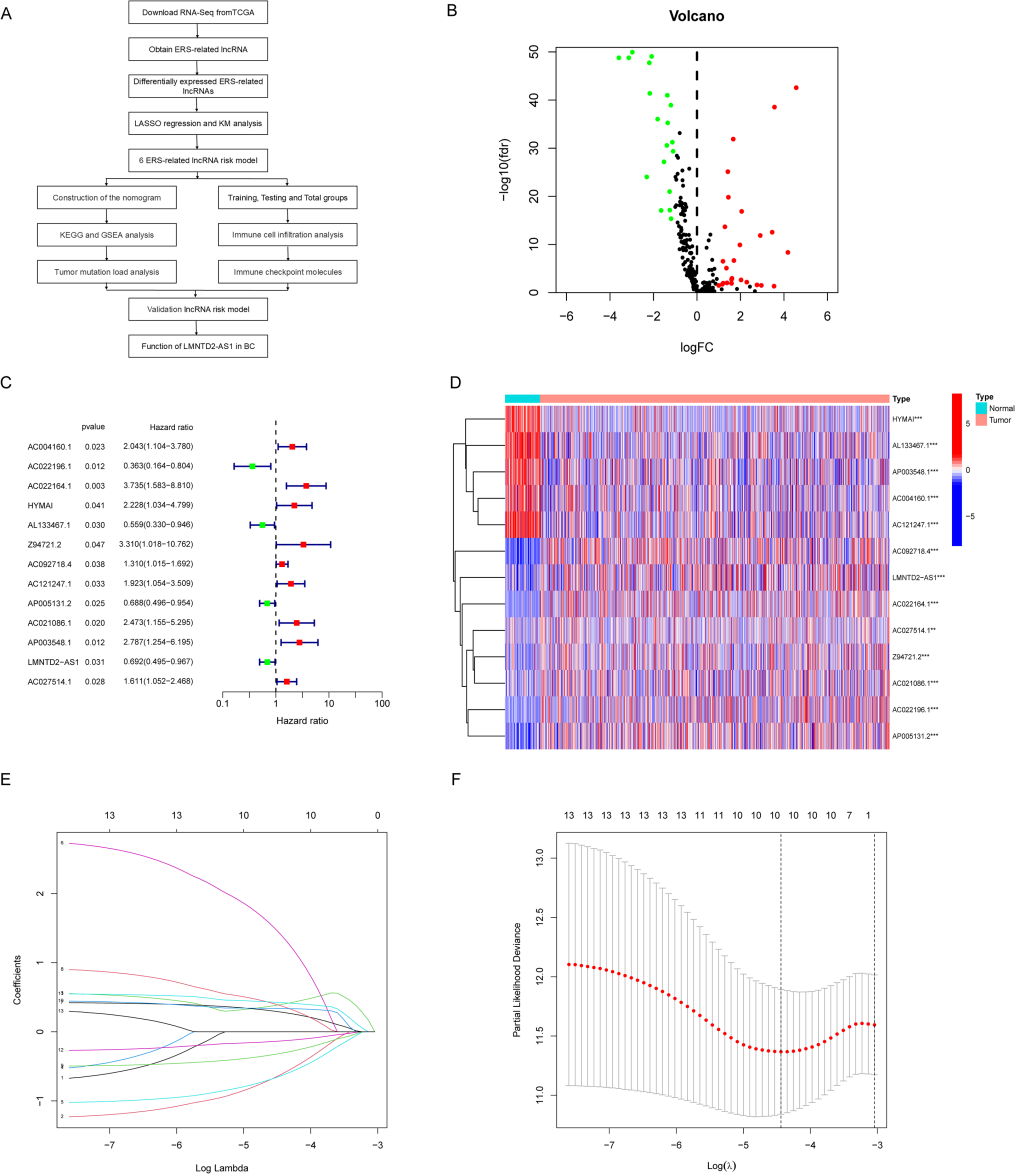
Identification of ERS-related lncRNAs with significant prognostic value in BC. **(A)** The flowchart of this study. **(B)** Volcano plot of ERS-related lncRNAs. Red dots indicate upregulated lncRNAs while the green dots indicate downregulated lncRNAs. **(C, D)** The forest and heatmap showed prognostic ERS-related lncRNAs by univariate Cox proportional hazards analysis. **(E)** LASSO coefficient profiles of the prognostic lncRNAs in BC. **(F)** Plots of cross-validation error rates.

**Figure 1A** presents the flowchart outlining the analytical steps of this study. We obtained RNA-Seq transcriptome profiles of 1,035 human breast tissue samples from TCGA, including 941 BC and 94 normal samples (https://portal.gdc.cancer.gov), and collected 295 ERS-related genes from published databases. Pearson correlation analysis identified 318 ERS-related lncRNAs, from which 45 differentially expressed ERS-related lncRNAs were detected between BC and normal tissues, as illustrated in the heatmap (**Supplementary Figure 1**). The volcano plot in **Figure 1B** further shows that among these 45 lncRNAs, 26 were upregulated and 19 were downregulated. Univariate regression analysis (**Figure 1C**) identified 13 ERS-related lncRNAs significantly associated with BC, and their expression in normal versus cancerous tissues is displayed in the heatmap in **Figure 1D**. Through LASSO regression analysis (**Figures 1E, F**), six lncRNAs most strongly associated with prognosis were identified: AC022196.1, LMNTD2-AS1, Z94721.2, AC092718.4, AC121247.1, and AP005131.2 (**Supplementary Table 1**). These six lncRNAs were subsequently selected to construct the prognostic model.

### Risk model efficacy and survival predictions

**Figure 2:**
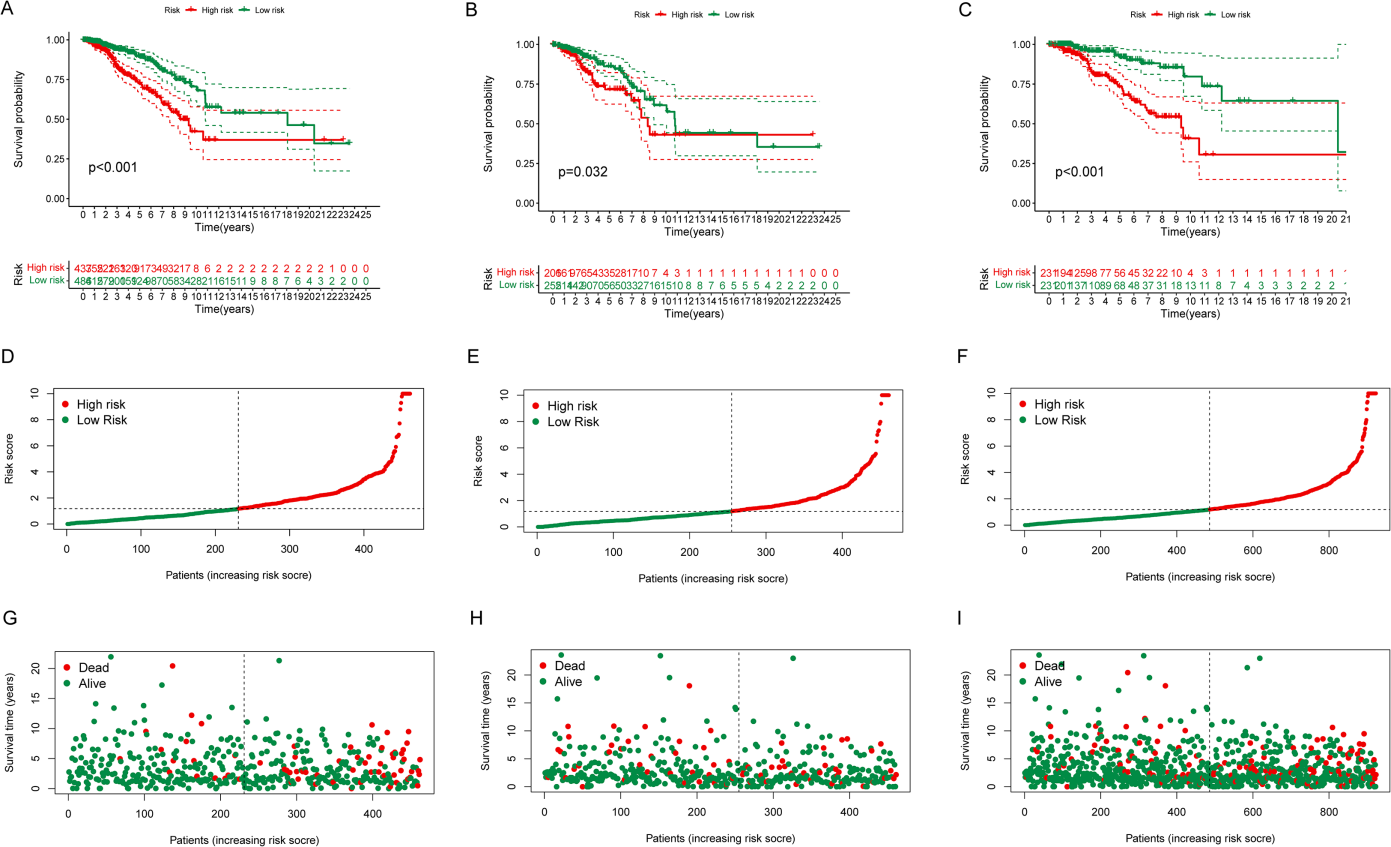
Risk score and overall survival of BC patients in the training and testing groups. **(A–C)** Kaplan-Meier survival curves show OS of BC patients in the high- and low-risk groups in the training **(A)**, testing **(B)** and all groups **(C)**. **(D–F)** Risk score distribution in the training **(D)**, testing **(E)**, and all groups **(F)**. **(G–I)** Survival status of BC patients in the training **(G)**, testing **(H)** and all groups **(I)**.

The refined risk model based on six ERS-related lncRNAs accurately predicted survival outcomes. Patients were stratified into high- and low-risk groups according to the median risk score derived from the six lncRNAs. Survival analysis revealed that BC patients in the high-risk group had significantly poorer OS compared with those in the low-risk group across the training, testing, and entire cohorts (**Figures 2A–C**). The distribution of risk scores and survival status based on the six-lncRNA signature is presented in **Figures 2D–F**. Consistently, elevated risk scores were associated with higher mortality, thereby validating the hypothesis that increased risk corresponds to worsened survival outcomes (**Figures 2G, I**).

**Figure 3:**
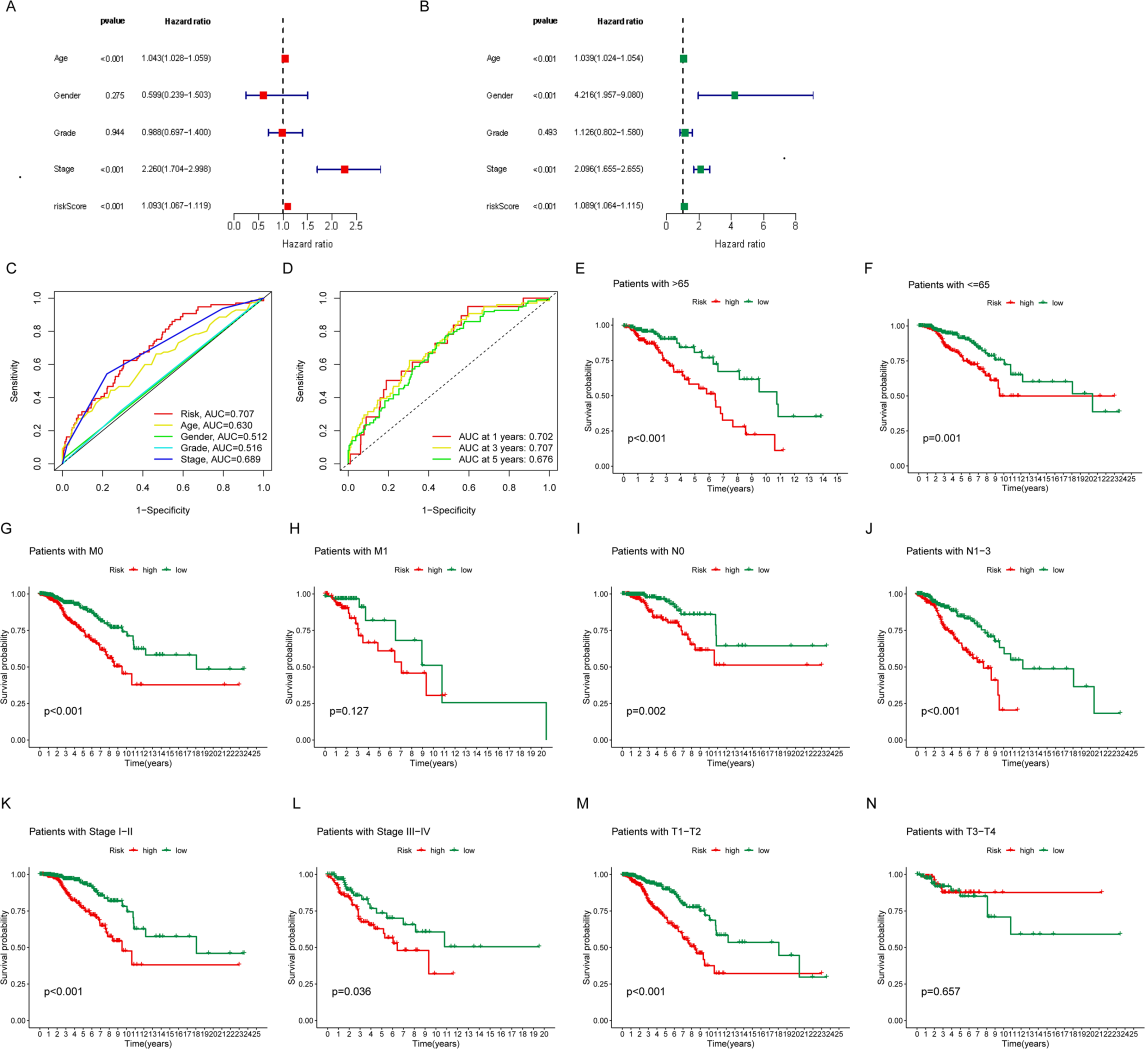
Relation between ERS-related lncRNA prognostic signature and clinical datas. **(A, B)** Univariate **(A)** and multivariate **(B)** Cox regression analyses of the risk score and clinical prognosis. **(C, D)** ROC and AUC curve constructed by risk model to predict patient survival. **(E–N)** Kaplan-Meier survival curves show the OS of BC patients from clinical datas including age, stage and TNM in two groups.

Univariate Cox regression analysis revealed that the OS of BC patients was significantly influenced by age, stage, and risk score (**Figure 3A**). Subsequent multivariate Cox regression further confirmed the risk score as an independent prognostic factor (**Figure 3B**). The predictive performance of the model was evaluated using ROC curves, which yielded an AUC of 0.707 for OS prediction (**Figure 3C**). Additionally, the AUC values for 1-, 3-, and 5-year survival were 0.702, 0.707, and 0.676, respectively, demonstrating a reasonable prognostic accuracy (**Figure 3D**). Kaplan-Meier survival analyses stratified by clinical variables further highlighted significant differences in OS between high- and low-risk groups across several clinical subgroups (**Figures 3E–N**).

### Construction of the nomogram for BC patients

**Figure 4:**
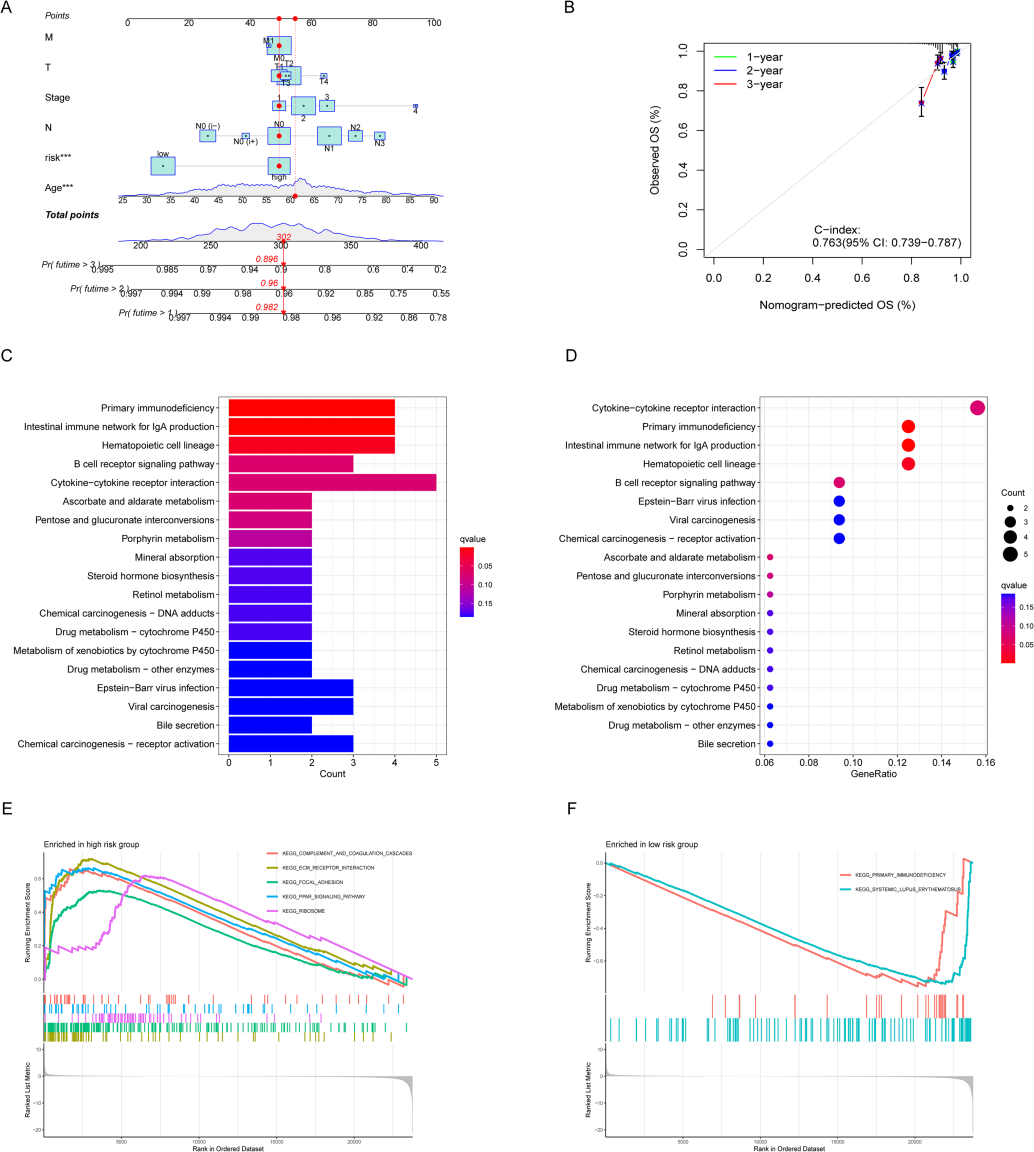
Establishment of the nomogram and functional enrichment analyses of the risk model. **(A)** The nomogram to predict overall survival of BC patients. **(B)** Thenomogram 1-,2-and 3-year OS calibration curve in BC patients. **(C, D)** Barplot and bubble plot of the Kyoto Encyclopedia of Genes and Genomes (KEGG). **(E, F)** GSEA pathway of DEGs enrichment analyses in BC patients.

To evaluate BC patients’ OS at 1-, 3-, and 5-year benchmarks, clinical variables were integrated with multivariate Cox regression analysis to generate risk scores, which were subsequently visualized through a nomogram (**Figure 4A**). Calibration analyses were then performed to assess predictive accuracy, and the results demonstrated strong agreement between predicted and observed OS probabilities (**Figure 4B**), with prediction accuracies of 0.982, 0.960, and 0.896 at 1, 3, and 5 years, respectively. Functional enrichment analysis further provided insights into the biological significance of the prognostic lncRNAs. KEGG pathway analysis (**Figures 4C, D**) suggested their potential involvement in immunodeficiency, IgA production in the intestinal immune system, hematopoietic cell lineage regulation, B cell receptor signaling, and various cytokine-receptor interactions. Additionally, GSEA revealed that the high-risk group was enriched in pathways including complement and coagulation cascades, ECM-receptor interaction, focal adhesion, PPAR signaling, and ribosomal biogenesis. In contrast, the low-risk group was primarily associated with primary immunodeficiency and systemic lupus erythematosus pathways (**Figures 4E, F**).

### Relevance and survival analysis of TMB

**Figure 5:**
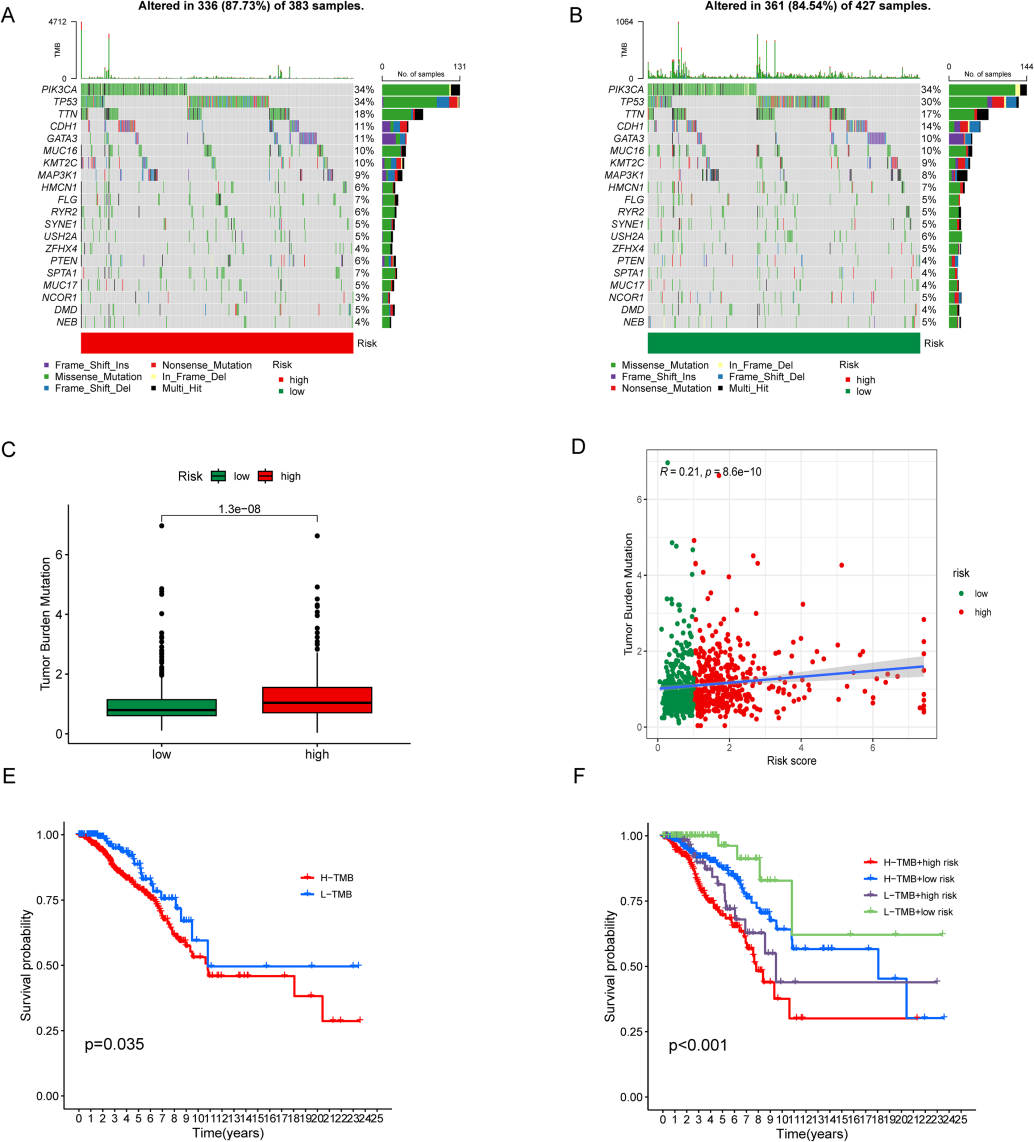
Relevance and survival analysis of tumor mutation load (TMB). **(A, B)** Waterfall plots of gene somatic mutations in high- and low-risk patients. **(C)** Boxplot show the differences of TMB in high- and low- risk groups. **(D)** Scatterplot show the relationship between TMB and the risk score. **(E)** Kaplan-Meier survival curves of BC patients between the H-TMB and L-TMB group respectively. **(F)** Kaplan-Meier survival curves of BC patients in H-TMB + high risk, H-TMB + low risk, L-TMB + high risk, and L-TMB + low risk group.

The waterfall plots revealed that the TMB was 87.73% in the high-risk group compared to 84.54% in the low-risk group (**Figures 5A, B**). Across both groups, PIK3CA, TP53, TTN, CDH1, and GATAD2A emerged as the five most frequently mutated genes, although their mutation frequencies varied between groups, with the exception of PIK3CA, which showed consistent rates. These findings suggest that ERS-related lncRNAs may be linked to established mutational events in BC. Moreover, patients in the high-risk group exhibited significantly higher TMB than those in the low-risk group (**Figure 5C**), and TMB levels were positively correlated with the risk score (**Figure 5D**). Survival analysis further demonstrated that patients with high TMB had poorer OS (**Figure 5E**). Notably, individuals characterized by both high TMB and high risk displayed the worst prognosis across the entire cohort (**Figure 5F**).

### Tumor microenvironment and immune cell infiltration analysis

**Figure 6:**
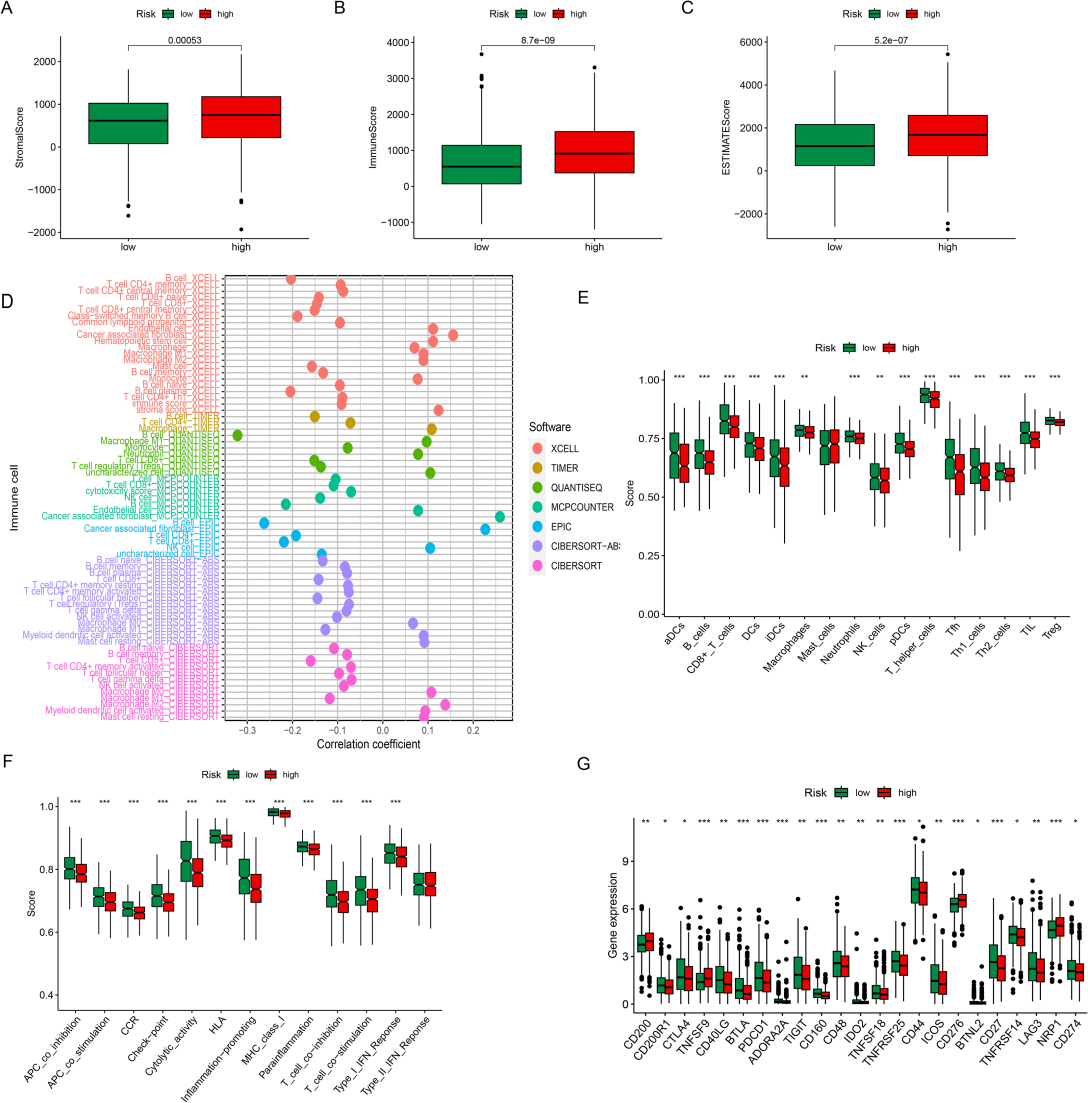
Tumor microenvironment and immune cell infiltration analysis. **(A–C)** Boxplots show the StromalScore **(A)**, ImmuneScore **(B)** and ESTIMATE Score **(C)** in the high- and low-risk groups. **(D)** Correlation analysis for immune infiltration cells in BC patients. **(E, F)** The score of the infiltrating immune cells (M) and immune cell subpopulations associated functions (N) in the high- and low-risk groups. **(G)** The expression of 23 immune checkpoint molecules between the two risk subgroups. *P < 0.05, **P < 0.01, ***P < 0.001.

Tumor purity was assessed by analyzing TME using the ESTIMATE algorithm, which generated immune, stromal, and ESTIMATE scores for each BC patient. All three scores were significantly higher in the high-risk group compared to the low-risk group (**Figures 6A–C**). Further immune cell infiltration analysis revealed that cancer-associated fibroblasts (XCELL), CD4^+^ T cells (TIMER), uncharacterized cells (QUANTISEQ), CD8^+^ T cells (EPIC), as well as M2 macrophages and resting mast cells (CIBERSORT) were positively correlated with the risk score. In contrast, memory CD4^+^ T cells (XCELL), B cells (QUANTISEQ), regulatory T cells (QUANTISEQ), naïve B cells (CIBERSORT), and CD8^+^ T cells (CIBERSORT) showed negative correlations with the risk score (**Figure 6D**). To further explore immune function, we examined the association between risk scores and immune cell subsets using ssGSEA. Except for mast cells, most immune-related functional cell types displayed significantly higher scores in the low-risk group (**Figure 6E**). Similarly, immune-related functions such as checkpoints, cytolytic activity(***P < 0.001), HLA antigen presentation(***P < 0.001), inflammation promotion(***P < 0.001), MHC I activity(***P < 0.001), T-cell co-inhibition/co-stimulation(***P < 0.001), parainflammation(***P < 0.001), and type I interferon responses(***P < 0.001) were all markedly enriched in low-risk patients compared with high-risk patients (**Figure 6F**). These results suggest that immune surveillance is enhanced in low-risk patients, which may contribute to their improved prognosis. Given these findings, we next investigated the relationship between risk score and immune checkpoint expression. High-risk patients demonstrated increased expression of CD200(**P < 0.01), TNFSF9(***P < 0.001), CD276(***P < 0.001), and NRP1(***P < 0.001), while the low-risk group exhibited higher expression of CD44(*P < 0.05), CD48(**P < 0.01), CD27(***P < 0.001), CTLA4(*P < 0.05), and PDCD1(***P < 0.001) (**Figure 6G**). Collectively, these results indicate distinct immune microenvironment characteristics between risk groups, underscoring the potential role of ERS-related lncRNAs in shaping tumor-immune interactions.

### Knockdown of LMNTD2-AS1 inhibits proliferation, migration and invasion of BC cells

**Figure 7:**
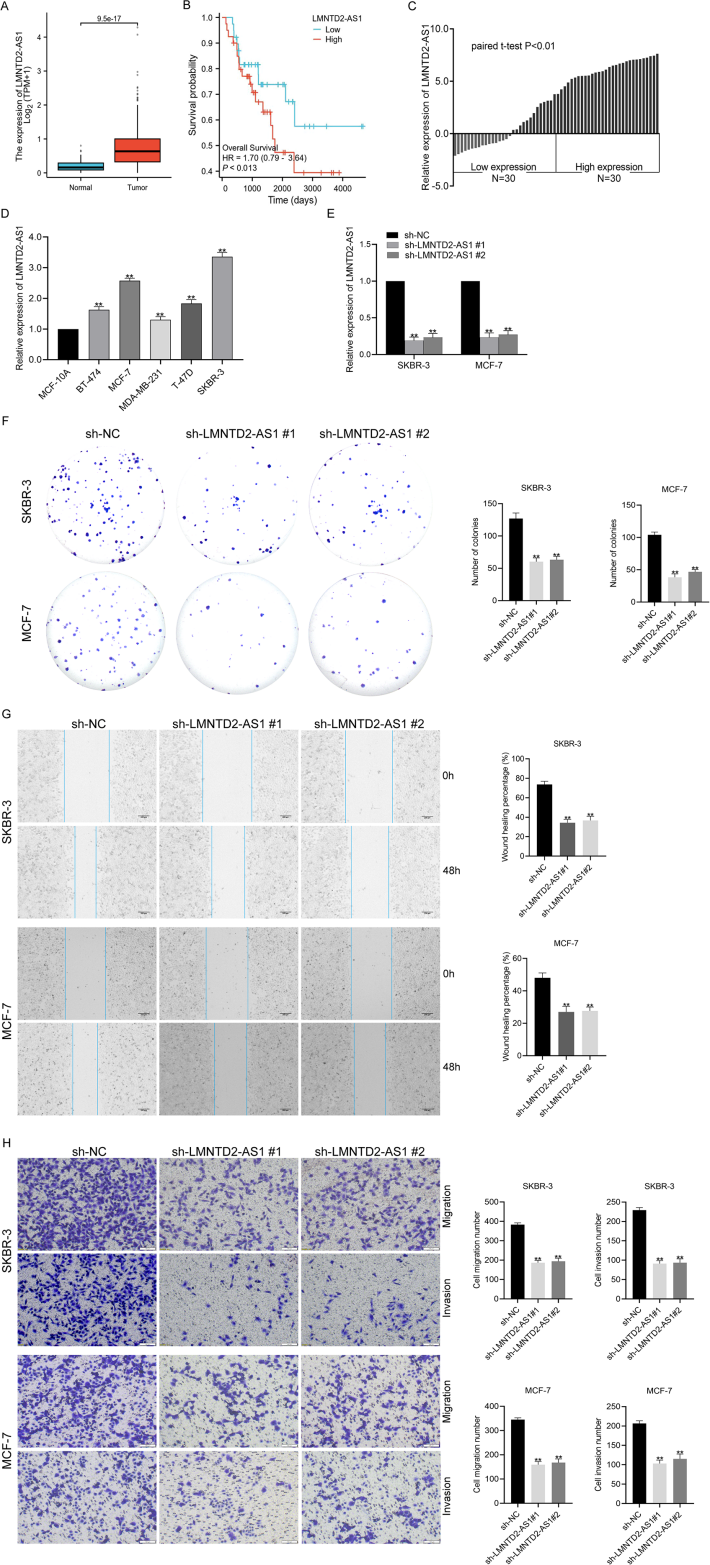
Knockdown of LMNTD2-AS1 inhibits proliferation, migration and invasion of breast cancer cells. **(A)** Analysis of LMNTD2-AS1 expression based on breast cancer transcriptome data from the TCGA database. **(B)** Association between LMNTD2-AS1 expression and the prognosis of breast cancer patients. **(C, D)** Evaluation of LMNTD2-AS1 expression in breast cancer tissues and cell lines by qRT-PCR. **(E)** Validation of sh-LMNTD2-AS1 knockdown efficiency in SKBR-3 and MCF-7 breast cancer cells using qRT-PCR. **(F)** Colony formation assay to assess the effect of LMNTD2-AS1 knockdown on the proliferative capacity of SKBR-3 and MCF-7 cells. **(G)** Wound-healing assay to examine changes in the migratory ability of SKBR-3 and MCF-7 cells upon LMNTD2-AS1 knockdown. **(H)** Transwell assay to confirm the role of LMNTD2-AS1 knockdown in regulating the migration and invasion abilities of SKBR-3 and MCF-7 cells. **P < 0.01.

To further explore the role of ERS-related lncRNAs in BC, we focused on LMNTD2-AS1, which has not been previously reported in the context of BC. Analysis of TCGA transcriptome data revealed that LMNTD2-AS1 expression was significantly elevated in BC tissues compared with normal controls (**Figure 7A**). Kaplan-Meier survival analysis demonstrated that high LMNTD2-AS1 expression was associated with poorer OS in BC patients (**Figure 7B**). To validate these findings, we performed qRT-PCR on 60 paired BC and adjacent non-tumor tissues, confirming that LMNTD2-AS1 was markedly upregulated in tumor samples (**Figure 7C**). Consistently, LMNTD2-AS1 expression was higher in multiple BC cell lines (BT-474, MCF-7, MDA-MB-231, T-47D, and SKBR-3) relative to the normal mammary epithelial cell line MCF10A (**Figure 7D**). Among these, SKBR-3 and MCF-7 exhibited the highest expression, and were therefore selected for functional assays. LMNTD2-AS1 knockdown was achieved using sh-LMNTD2-AS1 #1 and #2, with transfection efficiency validated by qRT-PCR after 48 hours (**Figure 7E**). Functional experiments demonstrated that LMNTD2-AS1 silencing markedly inhibited the proliferative capacity of SKBR-3 and MCF-7 cells, as shown by colony formation assays (**Figure 7F**). Wound healing assays revealed that LMNTD2-AS1 knockdown significantly reduced cell migratory ability after 48 hours (**Figure 7G**). Similarly, Transwell assays indicated that both migration and invasion capacities were impaired upon LMNTD2-AS1 suppression after 24 hours (**Figure 7H**). Collectively, these findings suggest that LMNTD2-AS1 promotes BC progression by enhancing tumor cell proliferation, migration, and invasion.

## Discussion

BC poses a significant health challenge worldwide, with current estimates indicating that 1 in 8 women will be diagnosed during their lifetime, making it the most prevalent cancer among women. The wide spectrum of clinical manifestations, coupled with its multifactorial etiology, renders prevention and treatment strategies highly complex and demanding (26, 27). Although substantial advances have been achieved in therapeutic interventions, many patients continue to suffer from a heavy disease burden, largely due to chemotherapy resistance and diminished quality of life. To improve outcomes, a deeper understanding of the molecular and cellular mechanisms driving BC progression, as well as factors influencing therapeutic response, is essential for the advancement of precision medicine. LncRNAs, typically ranging from 100 to 200 nucleotides in length, have emerged as crucial regulators of chromatin dynamics, gene expression, cellular proliferation, differentiation, and development (28). In BC, lncRNAs often display aberrant expression patterns and contribute to tumor initiation, progression, recurrence, and therapy resistance through multiple direct and indirect mechanisms, underscoring their potential as diagnostic and therapeutic targets. Moreover, they exert multilayered control over gene expression, via epigenetic modifications, transcriptional regulation, post-transcriptional processing, and translational control, making them key contributors to BC pathogenesis (29). Increasing evidence also highlights their prognostic value, particularly in risk prediction models, where lncRNA-based signatures have demonstrated clinical utility (30). For instance, Li et al. identified 39 ERS-related lncRNAs linked to poor prognosis in cutaneous melanoma (CM) and constructed a 10-lncRNA prognostic model, wherein patients in the low-risk group exhibited superior outcomes compared to their high-risk counterparts, with risk scores strongly correlating with immune cell infiltration in eight cases (31). Similarly, Huang et al. established a 6 ERS-related lncRNA signature in glioma that correlated positively with immune checkpoint activation and chemotherapy sensitivity, with functional analyses revealing enrichment in malignant processes, thereby suggesting their therapeutic and prognostic significance (32). In hepatocellular carcinoma (HCC), Zhang et al. developed an aberrant m6A-related lncRNA signature nomogram that effectively predicted prognosis and informed personalized treatment strategies, contributing further to precision oncology (33). In colon cancer, Liu et al. applied machine learning to construct an immune-related lncRNA signature (IRLS) that outperformed both clinical benchmarks and 109 published survival models (34). Furthermore, Zhang et al. proposed a necroptosis-associated lncRNA model capable of predicting immunotherapy sensitivity and prognosis in BC patients, offering new insights into individualized treatment approaches (35).

Through univariate Cox regression, Kaplan-Meier survival analysis, and LASSO regression, we identified six ERS-related lncRNAs, AC022196.1, LMNTD2-AS1, Z94721.2, AC092718.4, AC121247.1, and AP005131.2, as prognostic markers in BC. Li et al. reviewed AC022196.1 and recognized it as an immune-related lncRNA biomarker with potential application in BC immunotherapy within the framework of personalized medicine (36). Wu et al. demonstrated that LMNTD2-AS1 knockdown significantly suppressed migration, invasion, and proliferation of prostate cancer cells, effects that were reversed by FUS overexpression, suggesting that LMNTD2-AS1 may regulate immune cell infiltration and cancer progression through FUS-mediated NRF2 signaling interactions (37). Similarly, Chen et al. reported that silencing AC092718.4 inhibited lung adenocarcinoma (LUAD) cell growth while promoting apoptosis, thereby identifying it as a potential LUAD prognostic biomarker (38), whereas Yang et al. further established its prognostic relevance in BC as a disulfidptosis-related marker (39). Ge et al. highlighted AP005131.2 as a metabolism-associated lncRNA capable of predicting BC outcomes and guiding personalized therapy (40). Based on these findings, we constructed a risk prediction model incorporating the six ERS-related lncRNAs, which demonstrated strong prognostic accuracy across all cohorts, with high-risk BC patients exhibiting significantly reduced OS compared to low-risk patients. Higher risk scores were positively correlated with mortality and remained independent prognostic indicators in both univariate and multivariate Cox analyses. The nomogram we developed confirmed that patients classified as high-risk consistently exhibited poor prognostic outcomes. Functional enrichment analyses further revealed that these ERS-related lncRNAs were potentially involved in critical biological pathways. KEGG analysis indicated associations with intestinal immune networks for IgA production, hematopoietic cell lineage, B cell receptor signaling, and cytokine-cytokine receptor interaction, while GSEA demonstrated enrichment in complement and coagulation cascades, ECM-receptor interaction, focal adhesion, PPAR signaling, ribosome biogenesis, primary immunodeficiency, and systemic lupus erythematosus-related pathways. These results suggest that ERS-related lncRNAs may regulate BC progression by modulating immunological and metabolic processes, particularly through extracellular matrix (ECM) remodeling. Cancer cells regulate integrin receptors to control ECM dynamics, and progressive stiffening or degradation of the ECM has been linked to increased proliferation, migration, invasion, and angiogenesis (41). Focal adhesions influence nearly all aspects of cell behavior, while integrin signaling sustains tumor activity and promotes proliferation, survival, migration, invasion, and stemness (42). TMB analysis revealed that high-risk patients displayed markedly elevated TMB compared to low-risk individuals. Immune checkpoint analysis showed differential expression patterns: high-risk patients expressed elevated levels of CD200, TNFSF9, CD276, and NRP1, whereas low-risk patients primarily expressed CTLA4, CD44, CD48, CD27, and PDCD1. Overexpression of certain checkpoint molecules may suppress antitumor immune responses, thereby worsening prognosis. CD200, a novel cell surface glycoprotein, has been identified as a key immunomodulatory factor in BC through interactions with its receptor CD200R (43). Zhou et al. reported that CD276 (B7-H3) is overexpressed in over 60% of TNBC cases and is associated with angiogenesis, metastasis, and immune evasion (44). Furthermore, CTLA4+ T cells secrete S100A4, which induces a stem-like phenotype in TNBC cells (45), while CD44 has been recognized as a stem cell marker in BC that binds multiple ECM components, notably hyaluronic acid (HA), modifying tumor behavior through co-receptor interactions (46). Finally, TME analysis revealed that high-risk BC patients exhibited significantly higher stromal, immune, and estimate scores compared to their low-risk counterparts, reinforcing the clinical and biological impact of the identified ERS-related lncRNAs.

Based on the results of our analysis of six ERS-related lncRNAs, it appears that the role of LMNTD2-AS1 in BC has been relatively underexplored. To address this, we quantified LMNTD2-AS1 expression using qRT-PCR in both tumor tissues and adjacent non-tumor peritumoral tissues, observing a significant upregulation of LMNTD2-AS1 in tumor samples compared with their normal counterparts. Consistently, BC cell lines exhibited markedly higher LMNTD2-AS1 expression than the normal mammary epithelial cell line MCF10A. For functional knockdown experiments, we selected SKBR-3 and MCF-7 cells due to their relatively elevated LMNTD2-AS1 expression. Subsequent colony formation, wound-healing, and transwell assays revealed that silencing LMNTD2-AS1 substantially impaired the proliferative, migratory, and invasive capabilities of BC cells. Despite these findings, the precise molecular mechanisms through which LMNTD2-AS1 exerts its oncogenic influence in BC, as well as its potential interactions with other genetic and signaling pathways, remain to be clarified, warranting further investigation into its functional significance in BC progression.

In conclusion, we established a prognostic model based on six ERS-related lncRNAs that demonstrated robust predictive accuracy and significant clinical relevance for BC. This model effectively stratified patients by risk, enabling more precise identification of high-risk individuals with poorer outcomes. Beyond their prognostic utility, these lncRNAs, particularly LMNTD2-AS1, were shown to play functional roles in regulating BC cell migration and invasion, highlighting their potential as both biomarkers and therapeutic targets. Collectively, our findings provide valuable support for clinicians in refining BC prognosis and advancing personalized treatment strategies.

This study has several limitations. First, the data utilized were derived primarily from TCGA, which may introduce selection bias and limit the generalizability of the findings. Second, the precise molecular mechanisms through which LMNTD2-AS1 contributes to BC progression remain incompletely understood. Third, while our prognostic model incorporates multiple ERS-related lncRNAs, the functional roles of several of these lncRNAs require further experimental validation. Finally, although the model demonstrated strong predictive performance, its clinical utility must be confirmed through larger, multicenter, prospective studies to ensure robustness and broader applicability.

## Conclusion

In this study, we identified an ERS-related lncRNA prognostic model for BC using bioinformatics approaches and demonstrated its potential clinical significance. This retrospective analysis incorporated a pathway-centered exploration of BC outcomes through an integrated multi-omics framework, thereby offering deeper insights into disease mechanisms. The ERS-related lncRNA-based prognostic model represents an important advancement in the field, serving as a valuable tool for risk stratification, guiding personalized treatment strategies, and ultimately improving patient outcomes. Moreover, this work contributes to the broader understanding of BC biology and provides a foundation for future research aimed at refining prognostic models and developing novel therapeutic interventions.

## Data Availability

The datasets presented in this study can be found in online repositories. The names of the repository/repositories and accession number(s) can be found in the article/**Supplementary Material**.
