# Supplementary material for: Development and validation of a prognostic model based on endoplasmic reticulum stress-related lncRNAs in breast cancer
Source: Front Oncol. 2026 Jan 12;15:1613938. doi: 10.3389/fonc.2025.1613938 (PMC12832244; doi:10.3389/fonc.2025.1613938)
Supplement: Supplementary file 1 [file DataSheet1.docx]

**Supplementary Fig. 1 Heatmap of 45 differentially expressed ERS-related lncRNAs in BC.** Red indicates a high expression, while blue represents a low expression.


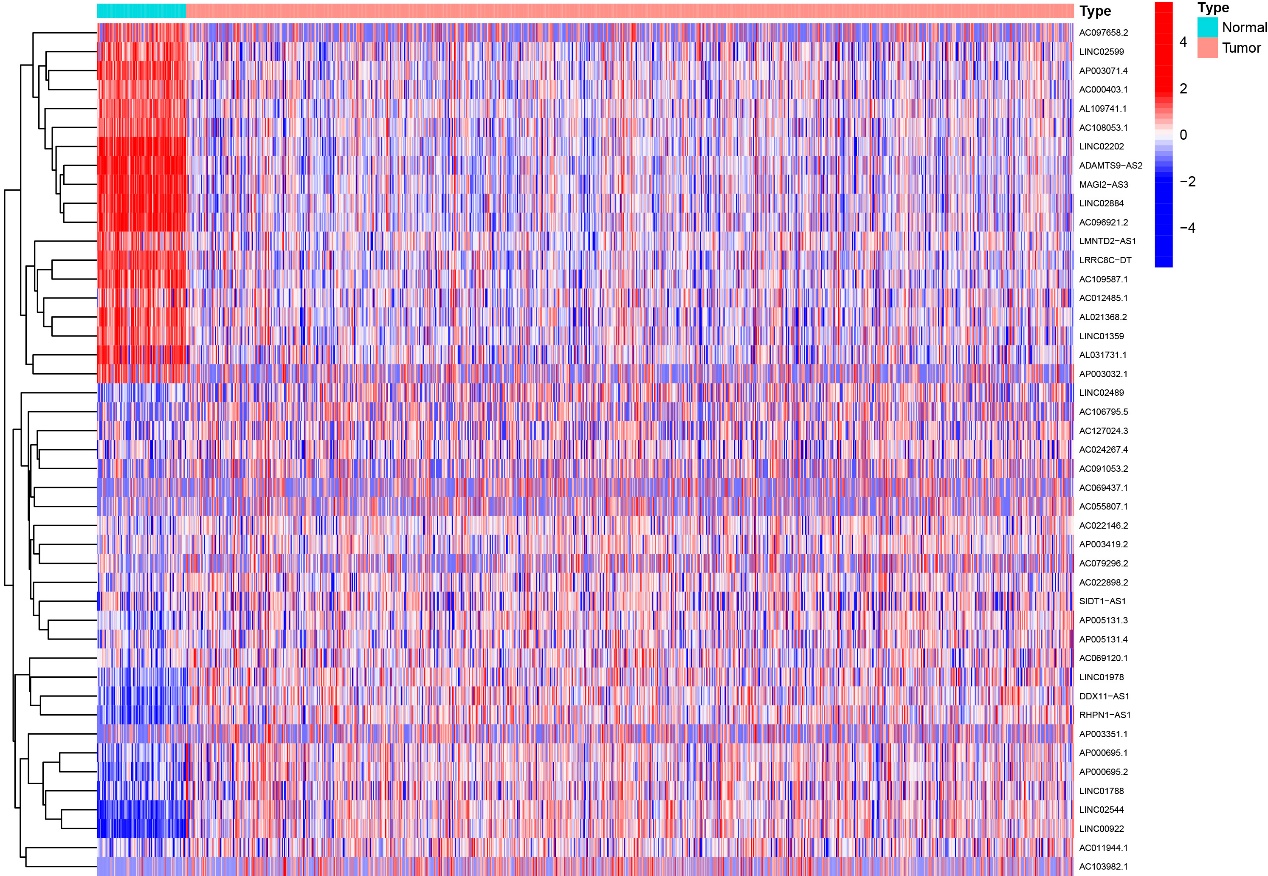


Supplementary Table 1. The prognostic significance of the 6 ERS-related lncRNAs signature.

ID Coef

AC022196.1 -1.07620381654464

LMNTD2-AS1 -1.98164874302256

Z94721.2 2.96310223224735

AC092718.4 0.450805708210458

AC121247.1 0.914387620053585

AP005131.2 -0.539003252287908
